# *qacA* is a key factor in heteroresistance to vancomycin in sequence type 5 methicillin-resistant *Staphylococcus aureus* isolates from pneumonia patients

**DOI:** 10.1128/spectrum.01963-25

**Published:** 2026-02-02

**Authors:** Kaiting Zhang, Lin Xi, Qiyu Bian, Yu Yin, Mengting Chen, Ping Yang, Zhouzhou Chen, Hailan Wu, Yongqiang Zhu, Huajun Zheng, Daijie Chen, Yaxin Fan, Jing Zhang

**Affiliations:** 1 Institute of Antibiotics, Key Laboratory of Clinical Pharmacology of Antibiotics, National Population and Family Planning Commission & National Clinical Research Center for Aging and Medicine, Huashan Hospital, Fudan University 159397, Shanghai, China; 2 School of Pharmacy, Shanghai Jiaotong University 12474, Shanghai, China; 3 Shanghai-MOST Key Laboratory of Health and Disease Genomics, Chinese National Human Genome Center at Shanghai and Shanghai Institute for Biomedical and Pharmaceutical Technologies 117748 , Shanghai, China; NHLS Tygerberg/Stellenbosch University, Cape Town, Western Cape, South Africa

**Keywords:** *qacA*, ST5, heterogeneous vancomycin-intermediate *S. aureus*, vancomycin

## Abstract

The presence of heterogeneous vancomycin-intermediate *Staphylococcus aureus* (hVISA) among methicillin-resistant *Staphylococcus aureus* (MRSA) strains has been associated with vancomycin treatment failure, especially in cases of MRSA pneumonia. This study aimed to analyze the molecular characteristics and risk factors for hVISA among MRSA strains isolated from pneumonia patients and to validate the most important risk factors. All the MRSA clinical isolates were collected in a multicenter, prospective, observational clinical trial from July 2012 to June 2020. The hVISA strains were verified via a modified population analysis profile–area under the curve method, and the prevalence of hVISA in hospitalized pneumonia patients was 53.5% (61/114). Sequence type 5 (ST5)-MRSA, the dominant hVISA strain, and ST764-MRSA, the dominant vancomycin-susceptible *S. aureus* (VSSA) strain, exhibited comparable incidences (27.2% vs 36.8%) but with different hVISA detection rates (90.3% vs 21.4%, *P* < 0.001). Furthermore, the efflux pump gene *qacA* was identified by multivariate logistic regression analysis as an independent significant predictor of hVISA occurrence in ST5-MRSA (*P* < 0.001). This association was confirmed when both the recipient ST5-MRSA and ST764-MRSA strains that acquired the *qacA*-borne plasmid exhibited phenotypic transformation from VSSA to hVISA. This study provides evidence that the acquisition of *qacA* by the VSSA strain is associated with the formation of hVISA in MRSA isolates from hospitalized pneumonia patients.

**IMPORTANCE:**

Methicillin-resistant *Staphylococcus aureus* (MRSA) is among the leading causes of pulmonary infections, and currently, vancomycin use remains an effective intervention. The irrational use of vancomycin has increased the prevalence of heterogeneous vancomycin-intermediate *Staphylococcus aureus* (hVISA)/vancomycin-intermediate *S. aureus* strains in pneumonia patients infected with MRSA, but the underlying molecular characteristics remain unknown. This study focused on the evidently high detection rates of hVISA strains from pneumonia patients from a prospective observational study in China and investigated the predictive risk factors for the dominant hVISA strain sequence type 5 (ST5)-MRSA. Multivariate logistic regression analysis of variables identified as significant for host, pathogen, and genetic characteristics revealed that qacA was a significant independent predictor of the development of hVISA in ST5-MRSA, which was confirmed by plasmid-based experiments. This study provides new insights and an improved understanding of the decreased vancomycin susceptibility of MRSA in pneumonia patients and provides evidence-based support for optimizing clinical treatment plans from an innovative perspective to maintain the efficacy of vancomycin.

**CLINICAL TRIALS:**

This study is registered with the China Clinical Trials Registry as ChiCTR-OPC-16007920 and ChiCTR-OPC-17012567 .

## INTRODUCTION

Methicillin-resistant *Staphylococcus aureus* (MRSA) is a prevalent nosocomial pathogen that causes severe conditions such as hospital-acquired pneumonia (HAP), bloodstream infections (BSIs), complicated skin and soft tissue infections, and infective endocarditis, resulting in significant morbidity and mortality (1). MRSA infection imposes a high and increasing burden on the healthcare system, ranking second in the assessment of public health burden and accounting for 19.2% of the total disability-adjusted life-years (2). The observed contradiction between the gradually declining prevalence of MRSA isolates in some regions, including China, and the increasing complexity of MRSA infection is partially a consequence of treatment advances and the ability of the pathogen to adapt to changing environments via alternative pathways (3, 4).

Vancomycin, which was introduced in the clinic more than 60 years ago, remains an effective intervention to treat MRSA infection. No vancomycin-resistant *S. aureus* strains have been identified in China, but an increasing prevalence of heterogeneous vancomycin-intermediate *Staphylococcus aureus* (hVISA) and vancomycin-intermediate *S. aureus* (VISA) strains has emerged under external selection pressure with wider utilization of and longer treatment with vancomycin. Multiple retrospective analyses have revealed that the hVISA phenotype is a potential risk factor for predicting vancomycin treatment failure and is responsible for considerable morbidity (5, 6).

Currently, the predominant hospital-acquired MRSA (HA-MRSA) genetic types globally are sequence type (ST) 239, ST36, and sequence type 5 (ST5), while ST239, ST241, and ST5 are the top three types in Asia (7). We previously identified ST5 as a risk factor for bacterial persistence in patients with MRSA pneumonia, and adults with ST5 MRSA infection showed worse clinical signs and symptom improvement than did those with ST764 infection (8, 9).

Notably, the epidemiologic features of hVISA/VISA strains were closely related to those of the ST5 and ST239 clones. A meta-analysis of hVISA epidemiology revealed that ST239 and ST5 are the most common epidemic genotypes of hVISA, with prevalence rates of 58.62% and 14.45%, respectively. Both are international HA-MRSA lineages prevalent in Asia, South America, and Eastern Europe, with Asia having the highest incidence (10).

It is likely that the features of hVISA are closely related to cell wall genetic mutations in ST5-MRSA, especially in two-component systems such as *walKR*, *vraSR*, and *graSR,* considering that the target of vancomycin is D-Ala-D-Lac, a material involved in bacterial peptidoglycan synthesis (11). However, the decreased susceptibility to antibiotics and the subsequent development of resistance often involve a multitude of diverse and interrelated mechanisms. Among them, efflux pumps may represent one of the most rapid and effective pathways under external stress, which has been well established in gram-negative bacteria; however, few studies have explored the genetic characteristics of hVISA strains in depth (12).

Retrospective studies have shown that the carriage of *qacA*, an *S. aureus*-specific efflux pump, is significantly associated with the hVISA phenotype in a single intensive care unit (ICU)-acquired MRSA strain, ST5 (13), but little is known about other MRSA infections in different regions or about this association. This study aimed to determine the molecular characteristics and risk factors for hVISA among MRSA strains isolated from pneumonia patients in China and then reveal and experimentally confirm the correlation between *qacA* and hVISA formation in ST5-MRSA.

## RESULTS

### Clinical MRSA isolates and the hVISA phenotype in pneumonia patients

All the MRSA clinical isolates were collected from a multicenter, prospective observational clinical trial conducted from July 2012 to June 2020. A total of 114 clinical MRSA strains were isolated from the sputum of Chinese patients diagnosed with MRSA pneumonia. Routine *in vitro* vancomycin susceptibility testing was performed using the agar dilution method. The MIC_50_ and MIC_90_ were both 1 mg/L, indicating that all 114 MRSA strains collected were sensitive to vancomycin.

**Fig 1:**
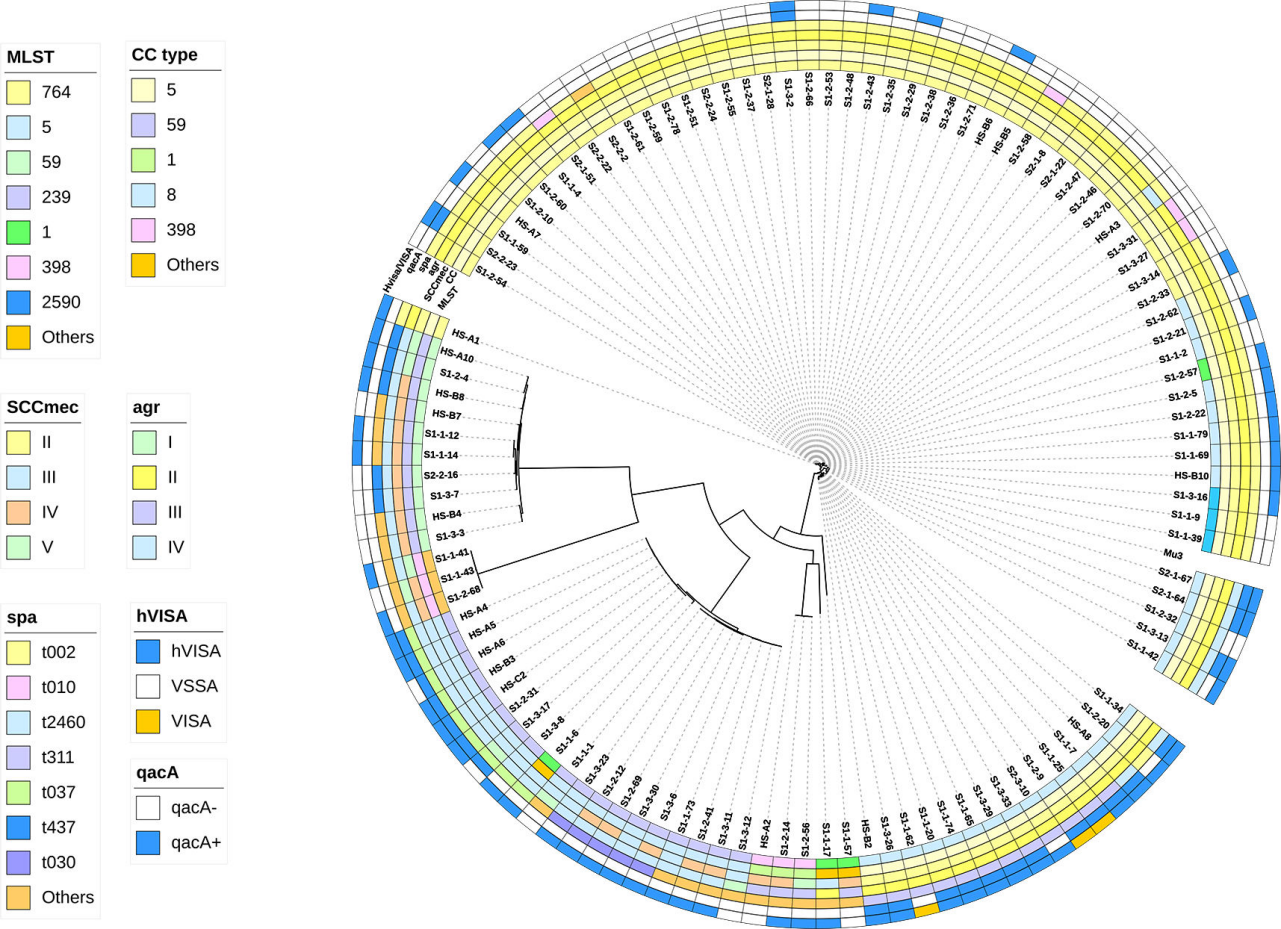
Phylogenetic tree of 114 MRSA strains with molecular typing, hVISA phenotype, and *qacA* carriage information.

hVISA strains were screened using the modified population analysis profile–area under the curve (PAP-AUC) method (14). Among these strains, 53.5% (61/114) hVISA and 2.6% (3/114) VISA strains were identified (Fig. 1). The significantly higher hVISA rate than that reported in historical data both globally and in China highlights the typical reduced vancomycin susceptibility of our MRSA strains. The MIC_50_ and MIC_90_ for hVISA/VISA were both 1 mg/L, whereas for the vancomycin-susceptible *S. aureus* (VSSA) strains, the MIC_50_ and MIC_90_ were 0.5 and 1.0 mg/L, respectively.

### Molecular typing of MRSA clinical isolates

Whole-genome sequencing (WGS) was performed on all 114 clinical MRSA isolates included in this study. A phylogenetic tree of 114 MRSA strains with CC, MLST, SCC*mec*, *agr*, and *spa* molecular typing is shown in Fig. 1.

CC5-SCC*mec*II-*agr*II-t002 was the dominant clone (48/114, 42.1%) in our study. CC5 was the most common epidemic subtype, accounting for 67.5% (77/114) of all the isolates, among which ST5 (31/77, 40.2%) and ST764 (42/77, 54.5%), differing in only one housekeeping gene, *aroE*, were the prevalent subtypes in the CC5 family. The SCC*mec*II type accounted for 66.7% (76/114) of all isolates, suggesting a hospital-related background for the MRSA strains. The *agr*II genotype, which is distributed mainly in *S. aureus* isolates that are moderately resistant to glycopeptides, accounted for 67.5% (77/114) of all the MRSA strains. *spa* typing revealed diversity, as the main class was t002 (50/114, 43.9%), followed by t311 (12/114, 10.5%) and t2460 (10/114, 8.8%).

Notably, ST5 was the most prevalent hVISA subtype (25/61, 41%), and all the VISA strains (3/3, 100%) identified belonged to the ST5 family. The main VSSA clone was ST764 (33/50, 66%). The vancomycin MIC assay results revealed that both the MIC_50_ and MIC_90_ of ST5-MRSA were 1 mg/L, whereas ST764-MRSA had an MIC_50_ of 0.5 mg/L and an MIC_90_ of 1.0 mg/L. The hVISA detection rate in ST5-MRSA was significantly greater than that in ST764-MRSA (90.3% vs 21.4%, *P* < 0.001).

The two phylogenetically close clonal types exhibited diverse phenotypes in terms of vancomycin susceptibility, indicating that further exploration of ST5-MRSA could reveal the mechanism underlying the high hVISA incidence via comparison against that of ST764-MRSA.

Genes encoding antimicrobial resistance factors were further investigated in the ST5-MRSA (*n* = 31) and ST764-MRSA (*n* = 42) clinical isolate groups via BLAST analysis against the Comprehensive Antibiotic Resistance Database (CARD) (15) (Table S1). In total, 44 antimicrobial resistance genes were identified from the ST5-MRSA and ST764-MRSA strains. Twenty-five genes, including *mecA*, *mecR*, *tet(38)*, *parC*, *gyrA*, and *arlR*, were carried by all 73 strains, whereas six genes [*norA*, *rpsJ*, *mecl*, *ant(9′)-la*, *uhpt*, and *fosB3*] presented marginal differences between the two groups, with detection rates greater than 90%, indicating the high potential for resistance of the MRSA strains to multiple antimicrobial agents. Three genes (*mupA*, *fusC*, and *cat7A*) presented a low prevalence (<10%) in both groups. Notably, the prevalence of six genes [*qacA*, *blaZ*, *ant(4*′*)*, *lunA*, *tetM*, and *mecR1*] significantly differed between the ST5-MRSA and ST764-MRSA groups (Table 1). *qacA*, *blaZ*, *ant(4*′*)*, and *lunA* were the beneficial genes in ST5-MRSA, whereas *mecR1* and *tetM* were the beneficial genes in ST764-MRSA.

**TABLE 1:** Antimicrobial resistance-related gene carriage in ST5-MRSA and ST764-MRSA

Gene	Annotation	Total (*n* = 73)	ST5-MRSA (*n* = 31)	ST764-MRSA (*n* = 42)
*mecR1*	Signal transduction gene, penam antibiotic target replacement	63 (86.3%)	23 (74.2%)	40 (95.2%)
*tetM*	Tetracycline-resistant ribosomal protection target	58 (79.5%)	16 (51.6%)	42 (100%)
*ant(4*'*)*	Aminoglycoside antibiotic inactivation	21 (28.8%)	17 (54.8%)	4 (9.5%)
*blaZ*	β-Lactamase inactivation	20 (27.4%)	20 (64.5%)	0 (0%)
*qacA*	Major facilitator superfamily antibiotic efflux pump	20 (27.4%)	18 (58.1%)	2 (4.8%)
*lunA*	Lincosamide-nucleotidyl transferase antibiotic inactivation	10 (13.7%)	10 (32.3%)	0 (0%)

### Clinical characteristics of patients with ST5- and ST764-MRSA pneumonia

The clinical characteristics of the 73 enrolled patients with pneumonia caused by infection with ST5-MRSA or ST764-MRSA are summarized in Table 2. More than half of the patients were male (63%), with a median age of 72 (57–81) years. Most of the patients (68.5%) had a history of intensive care unit (ICU) admission, with a median stay of 24 (7–38) days. Histories of cardiovascular disease and cerebral apoplexy were found in 34.2% and 38.4% of the pneumonia patients, respectively. Invasive procedures such as venous catheter implantation, endotracheal intubation, and tracheotomy were implemented in 71.2%, 35.6% and 28.8% of the patients, respectively. The MIC_90_ of vancomycin for the 73 ST5 and ST764 MRSA isolates was 1 mg/L, while the MIC_50_ was higher for the ST5 strain (1 mg/L) than for the ST764 strain (0.5 mg/L).

**TABLE 2:** Comparison of host, vancomycin treatment, and bacterial molecular typing between ST5-MRSA and ST764-MRSA^*b*^

Characteristic^*a*^	Total (*n* = 73)	ST5-MRSA (*n* = 31)	ST764-MRSA (*n* = 42)	*P* value^*c*^
Demographic				
Age (years)	72 (57–81)	63 (50–75)	75 (60–83)	**0.010**
Sex, male	46 (63.0%)	17 (54.8%)	29 (69.0%)	0.214
Weight (kg)	60 (55–70)	60 (54–70)	60 (55–70)	0.842
BMI (kg/m^2^)	21.7 (19.5–23.6)	22.2 (19.8–23.0)	21.5 (19.0–24.0)	0.843
ICU				
ICU admission	50 (68.5%)	20 (64.5%)	30 (71.4%)	0.530
ICU stay (days)	24 (7–38)	27 (13–40)	21 (2–35)	0.386
Underlying disease/condition				
Cardiovascular disease	25 (34.2%)	7 (22.6%)	18 (42.9%)	0.071
Diabetes	11 (15.1%)	3 (9.7%)	8 (19.0%)	0.438
Cerebral apoplexy	28 (38.4%)	6 (19.4%)	22 (52.4%)	**0.004**
Dialysis	2 (2.7%)	1 (3.2%)	1 (2.4%)	>0.999
COPD	4 (5.5%)	1 (3.2%)	3 (7.1%)	0.836
Autoimmune disease	6 (8.2%)	2 (6.5%)	4 (9.5%)	0.967
Trauma	12 (16.4%)	6 (19.4%)	6 (14.3%)	0.564
Solid tumor	1 (1.4%)	1 (3.2%)	0 (0%)	0.425
Operation	30 (41.1%)	16 (51.6%)	14 (33.3%)	0.117
Implant				
Venous catheter	52 (71.2%)	20 (64.5%)	32 (76.2%)	0.276
Endotracheal intubation	26 (35.6%)	16 (51.6%)	10 (23.8%)	**0.014**
Tracheotomy	21 (28.8%)	16 (51.6%)	5 (11.9%)	**<0.001**
PD				
MIC_50_ (mg/L)	1	1	0.5	NA
MIC_90_ (mg/L)	1	1	1	NA
Molecular typing				
SCC*mec*II	72 (98.6%)	30 (96.8%)	42 (100%)	0.425
*agr*II	72 (98.6%)	31 (100%)	41 (97.6%)	>0.999
*spa*-t002	46 (63.0%)	9 (29.0%)	37 (88.1%)	**<0.001**

^*a*^ *agr*, accessory gene regulator; BMI, body mass index; COPD, chronic obstructive pulmonary disease; ICU, intensive care unit; NA, not applicable; PD, pharmacodynamics; SCC*mec*, staphylococcal cassette chromosome *mec*;* spa*, *staphylococcal* protein A.

^*b*^ Continuous variables are expressed as the median (interquartile range), and categorical variables are summarized as the number of cases (%).

^*c*^ Boldfaced values indicate *P* values less than 0.05 and are considered statistically significant.

### Predictive factors for hVISA occurrence in ST5-MRSA compared with that in ST764-MRSA

The ST5-MRSA and ST764-MRSA clinical isolate groups were further compared in terms of hosts, vancomycin pharmacodynamics (PD) and bacterial factors. As shown in Table 2, in terms of clinical features, patients in the ST5-MRSA group were younger (63 vs 75 years) and more likely to have undergone endotracheal intubation (51.6% vs 23.8%) and tracheotomy (51.6% vs 11.9%) than those in the ST764-MRSA group were; moreover, patients infected with ST764-MRSA had a greater incidence of cerebral apoplexy (52.4% vs 19.4%). In addition, the *spa*-t002 genotype was the dominant variant in ST764-MRSA over ST5-MRSA (88.1% vs 29.0%).

**Fig 2:**
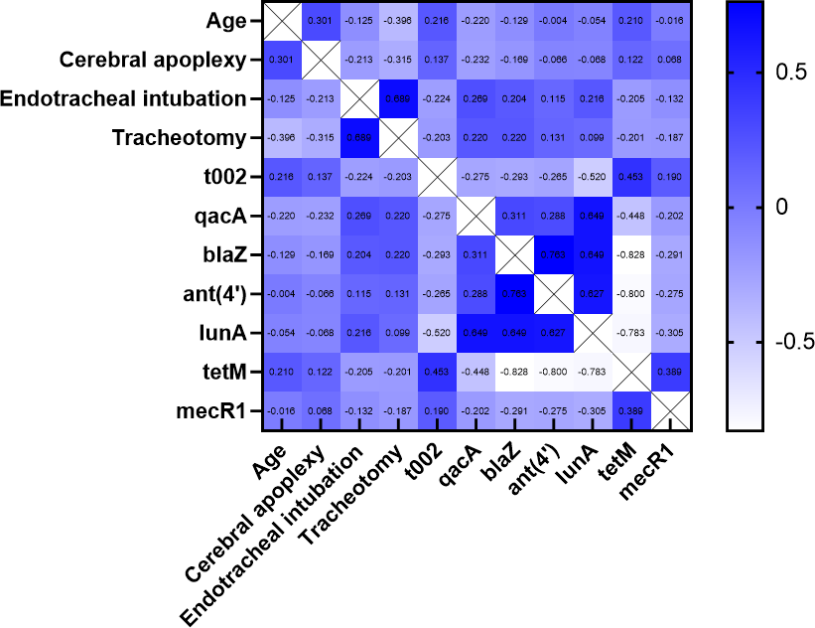
Correlation analysis of significant variables related to the host, vancomycin treatment, and genotype.

Correlation analysis was performed on all significant single variables from the host, vancomycin PD, and genotyping comparisons between the ST5-MRSA and ST764-MRSA strains (Fig. 2) to assist in variable selection for subsequent multivariate analysis. Seven variables, including age, cerebral apoplexy, endotracheal intubation, *spa*-t002, *qacA*, *ant(4*′*)* and *mecR1*, were included in the multivariate logistic regression analysis of hVISA occurrence.

Finally, multivariate analysis (Table 3) revealed that the *qacA* gene was an independent predictor of hVISA occurrence in ST5-MRSA strains (aOR, 48.323; 95% CI, 6.004–366.565; *P* < 0.001). The Hosmer–Lemeshow goodness-of-fit test revealed that the model fit well (*P* > 0.05).

**TABLE 3:** Multivariate logistic regression model of risk factors associated with hVISA occurrence in ST5-MRSA

Risk factor^*a*^	Univariate analysis (OR [95% CI])	Multivariate analysis	
		aOR (95% CI)	*P* value
Age (years)	0.834 (0.924–0.988)		
Cerebral apoplexy	0.423 (0.109–1.646)		
Endotracheal intubation	3.010 (0.731–12.398)		
*spa-*t002	0.152 (0.051–0.454)		
*qacA*	36.944 (4.571–289.601)	48.323 (6.004–366.565)	<0.001
*ant(4*′*)*	3.409 (1.140–10.190)		
*mecR1*	0.089 (0.011–0.744)		

^*a*^ aOR, adjusted odd ratio; CI, confidence interval.

### Carriage of the efflux pump gene *qacA* mediated hVISA occurrence in ST5-MRSA isolates

The *qacA* carriage rates of our MRSA isolates are presented in Fig. 1. A total of 58.1% (18/31) of the ST5-MRSA strains carried the *qacA* gene, and the *spa* subtypes t311 and t2460 accounted for 32.3% (10/31) and 25.8% (8/31) of the strains, respectively. Approximately half (13/31) of the ST5-MRSA strains were *qacA*(−), and most of the *qacA*(−)-ST5-hVISA strains belonged to the t002 subtype (29.0%, 9/31). The transmission of *qacA* was clearly lineage dependent, and compared with ST5-t002, ST5-t311 and ST5-t2460 were more advantageous clones.

The ability of *qacA* carriage in ST5-MRSA to mediate phenotypic transformation from VSSA to hVISA was verified by observing the phenotypic changes after the electroporation of *qacA*-borne plasmids into specific recipient strains of the ST5 (S1-2-32) and ST764 (S1-2-46) strains (Table 4). Polymerase chain reaction (PCR) experiments confirmed that all the plasmids were successfully transferred into the recipient strains. An *in vitro* vancomycin susceptibility test revealed no significant change in the MIC values for each strain that acquired the *qacA*-harboring plasmid. After electroporation with a plasmid carrying *qacA*, the ST5-MRSA (S1-2-32*qacA*) and ST764-MRSA (S1-2-46*qacA*) strains clearly exhibited an hVISA phenotype, as confirmed by the modified PAP-AUC method.

**TABLE 4:** hVISA phenotype of the wild-type and transformed ST5 and ST764 strains

ST type	Strain serial number	Vancomycin MIC (mg/L)	AUC_test_	AUC_test_/AUC_Mu3_	hVISA/VSSA^*a*^
ST5	S1-2-32	0.5	22.51	0.8	VSSA
	S1-2-32*qacA*	0.5	26.69	1.0	hVISA
ST764	S1-2-46	0.5	22.06	0.8	VSSA
	S1-2-46*qacA*	0.5	28.18	1.1	hVISA
Mu3 (positive control)		0.5	26.93		hVISA

^*a*^ hVISA: AUC_test_/AUC_Mu3_∈(0.9–1.3); VSSA: AUC_test_/AUC_Mu3_ <0.9.

## DISCUSSION

hVISA was first isolated in 1997 from the pathogen Mu3 collected from sputum samples of pneumonia patients (15), after which emerging cases were reported in various countries and regions. A meta-analysis of hVISA/VISA epidemiology demonstrated overall prevalence rates of 6.05% and 3.01% for hVISA and VISA, respectively (10). Our analysis utilized clinical MRSA strains collected from MRSA-infected HAP patients and revealed a significantly greater incidence of hVISA (53.5%, 61/114) via PAP-AUC, which was more than triple the combined prevalence rate for hVISA in mainland China (15.78%) (16–19). Because MRSA infection occurrence, especially healthcare-associated infections, is increased in Asian countries (20), this has led to more frequent use of vancomycin, which poses selective pressure on endemic MRSA strains. An investigation of the mechanisms underlying the emergence of regional hVISA has significant clinical implications for treatment with vancomycin.

The genetic prevalence of hVISA is dominated by ST239 (58.62%) and ST5 (14.45%) globally, and almost half of hVISA strains are SCC*mec*II-type strains (48.16%) (10). Our MRSA isolates presented the reverse genetic profile of hVISA, with ST5 (41.0%, 25/61) being the most common epidemic variant, followed by ST239 (23.0%, 14/61), whereas SCC*mec*II (63.9%, 39/61) accounted for more than half of all the hVISA strains. This SCC*mec*II majority was in line with the healthcare background of our MRSA strains isolated from a nosocomial environment, but the unique and subtle dominance of ST5 over ST239 in our hVISA strains from hospitalized pneumonia patients made further investigation of the underlying factors worthwhile.

Many studies have focused on describing the epidemiology and molecular characteristics of hVISA/VISA strains directly, whereas few have focused on choosing a suitable reference to perform in-depth comparisons; such a reference would ideally be phylogenetically similar but phenotypically different from the typical hVISA clone. As a single-locus variant of ST5-MRSA, the ST764 clone diverged from the ST5 lineage via a single genetic mutation and is a hybrid variant with community-associated features. ST764 has gradually become prevalent among nosocomial MRSA infections in Asian countries in recent decades (21, 22). A recent study reported that ST5/ST764-SCC*mec*II was the most common type among MRSA-associated BSIs in southern China (23). Interestingly, our study identified ST5/ST764-SCC*mec*II as the dominant clone in HAP MRSA infections (63.2%, 72/114), and the similar proportions of the ST5 (27.2%, 31/114) and ST764 (36.8%, 42/114) clonotypes indicate possible coevolution, given their genetic relatedness. In contrast to the high frequency of ST5 among hVISA strains, ST764 was the representative lineage among VSSA strains. The different MIC_50_ values of ST5-MRSA (MIC_50_ = 1 mg/L) and ST764-MRSA (MIC_50_ = 0.5 mg/L) also indicated their phenotypic diversity in terms of hVISA, as changing vancomycin MICs directly impact hVISA rates, because the proportion of hVISA isolates increases with increasing vancomycin MICs, even within the susceptible range (24). Thus, the ST5-MRSA and ST764-MRSA strain groups represent a suitable match to further elucidate the mechanism underlying the high hVISA frequency in ST5-MRSA.

Depending on the antibiotic challenge imposed, efflux could be the fastest-acting and most effective resistance mechanism in the bacterial repertoire of stress responses and is not only commonly observed in gram-negative species but also a significant contributing factor to gram-positive resistance. Efflux pumps can dynamically transport not only clinically relevant antimicrobial agents but also environmental biocides and disinfectants that colonize the healthcare environment; these multiple structurally unrelated compounds result in a multidrug resistance (MDR) phenotype (12, 25). Compared with that of ST764-MRSA, the efflux pump gene *qacA* was identified in our study as a distinguishable gene carried by ST5-MRSA as a predictor of hVISA formation. The *qacA* gene encodes the QacA protein, which is an *S. aureus*-specific MDR efflux pump that uses a proton motive force-dependent antiport mechanism to drive the export of various cationic and lipophilic compounds, such as chlorhexidine, a commonly used decolonization agent for MRSA in hospitals, to mediate *S. aureus* resistance or reduce susceptibility to many clinical antibiotics (26). QacA genes are plasmid encoded and thus are relatively easily transferred horizontally between species and clonotypes. The expression of plasmid-based genes is usually sufficient for relevant resistance to be observed without the need for additional mutations because of the multicopy nature of these genetic elements (25).

Multiple studies have indicated that clinical strains carrying the *qacA* gene are predominantly hospital-associated pathogens such as ST5-MRSA (27–29), which is consistent with our finding that the ST5 type accounted for a majority (66.6%, 18/27) of the *qacA*(+)-MRSA strains. Interestingly, all the *qacA*(+)-ST5-MRSA strains were hVISA/VISA strains, which have not been previously reported. Although little is known about whether *qacA* is associated with reduced vancomycin susceptibility or with ST5-MRSA, experiments and analyses have been conducted to investigate this aspect. After successful electroporation of the *qacA*-borne plasmid extracted from the *qacA*(+)-ST5-hVISA strain into the *qacA*(−)-ST5-VSSA and *qacA*(−)-ST764-VSSA strains, the hVISA phenotype was observed in the electroporated strains. In addition to the selective advantage for ST5-MRSA in hospital environments, *qacA* carriage also provided it with reduced vancomycin susceptibility as a significant contributor to the transformation of VSSA to hVISA.

Moreover, one *qacA*(−)-ST764-VSSA strain was also selected for the electroporation experiment, in addition to the *qacA*(−)-ST5-VSSA strain, as we aimed to explore whether *qacA*-mediated hVISA formation is lineage dependent. In almost half of the *qacA*(−)-VSSA strain group (41.2%, 47/114), the abundance of the ST764 lineage was more than 10 times greater (68.1%, 32/47) than that of the ST5 lineage (6.4%, 3/47). The successful phenotypic change in the *qacA*(−)-ST764-VSSA strain indicated that the generality of *qacA* carriage for hVISA formation was not restricted to ST5-MRSA but was applicable to other genotypes. The significantly greater carriage of *qacA* in our ST5-MRSA isolates may have contributed to the higher hVISA detection rate than that in ST-764. With MRSA infection expanding from the nosocomial environment to the community environment, the association of *qacA* with hVISA formation identified from hospital isolates of ST5-MRSA also has clinical implications for community-characterized MRSA strains, such as ST764-MRSA strains.

Notably, an association of *qacA* with the hVISA phenotype was previously reported by Cho et al. (13) among MRSA strains in the ICU during decolonization with chlorhexidine. Our study further revealed a similar significant correlation among MRSA isolates from pneumonia patients. However, similar conclusions have been drawn from different ratiocinations, as our research focused on the significantly higher hVISA rate observed in pneumonia patients infected with MRSA, and stepwise analysis revealed that *qacA* is a risk factor for hVISA formation in ST5-MRSA. However, their work focused directly on the spread of *qacA* in patients undergoing chlorhexidine-based MRSA decolonization and revealed its potential link to clinical characteristics, including the hVISA phenotype. Moreover, associations may be nonspecifically detected, especially from clinical MRSA isolates with complex backgrounds and many confounding factors. Thus, our work further confirmed this association as transferable and reproducible in terms of phenotype change to hVISA in experimentally electroporated ST5 and ST764 MRSA strains after the acquisition of the *qacA*-borne plasmid. Our MRSA strains were collected from 10 hospitals nationwide, which is geographically representative of MRSA epidemiology in China and in line with the findings from a single ICU in Korea, with some commonalities in the pattern in which *qacA* affects hVISA occurrence in Asia.

Admittedly, our analysis has several limitations. The molecular mechanisms underlying the development of hVISA strains are interactive and confounding, and a comprehensive assessment could still be applied to further understand the correlation. However, we believe that the findings do not undercut the evident association of *qacA* with hVISA formation in the ST5-MRSA strains identified in our study. Further work is needed to explore the possible mechanism underlying this phenomenon in terms of *qacA* expression, *qacA*-mediated vancomycin recognition, and extrusion to confirm the identified correlation.

In summary, a higher frequency of hVISA than typically recorded was observed in clinical MRSA pneumonia isolates, with ST5 being the dominant hVISA strain and ST764 being the dominant VSSA strain. The efflux pump gene *qacA* was identified as a significant predictor of hVISA formation in the ST5-MRSA strains. *qacA* carriage is lineage-specific in that the ST5-t311 and ST5-t2460 MRSA strains are advantageous clonal variants over ST5-t002 in terms of *qacA* carriage. The *qacA*-borne plasmid promoted the transformation of ST5-MRSA from VSSA to hVISA as an adaptive response that contributed to reduced vancomycin susceptibility during infection in healthcare settings.

Further research on *qacA* in ST5-MRSA is warranted in an effort to improve our understanding of how these systems interact with vancomycin. This knowledge may inform the development of means to postpone the occurrence of vancomycin resistance during MRSA infection in pneumonia patients and provide innovative perspectives on the effectiveness of vancomycin.

## MATERIALS AND METHODS

### Bacterial isolates

This study was conducted at 10 teaching hospitals from July 2012 to June 2020 and used a database containing data collected from two multicenter, prospective observational clinical studies.

All clinical isolates of *S. aureus* were collected, and identical strains from the same patient were excluded. The MICs of oxacillin and vancomycin were determined by the agar dilution method at a CHINET microbiology laboratory. Strains that grew at an oxacillin concentration of ≥4 mg/L were identified as MRSA.

### hVISA screening

A modified PAP-AUC method with vancomycin concentrations of 0.5, 1.0, 2.0, 2.5, 4.0, and 8.0 mg/L was adopted to screen for hVISA strains (14). Tested isolates with an AUC ratio against the control strain (Mu3, ATCC 700698) greater than 1.3 were defined as VISA strains; those with ratios between 0.9 and 1.3 were defined as hVISA strains; and those with ratios less than 0.9 were defined as VSSA strains.

### WGS and genotyping

DNA from MRSA strains was extracted with a Takara MiniBEST Bacteria Genomic DNA Extraction Kit v.3.0 (Takara Biomedical Technology Co., Ltd., Beijing, China). A 300 bp double-ended library was generated using the NEXTflex DNA Sequencing Kit (Bio Scientific, USA) and sequenced on an Illumina X10 platform (Illumina, San Diego, CA, USA) via 2 × 150 bp paired-end sequencing.

The sequence data of seven housekeeping genes (*arcC*, *aroE*, *glpF*, *gmk*, *pta*, *tpi*, and *yqiL*) were analyzed for multifocal sequence typing according to the PubMLST database (https://pubmlst.org/). Six of seven identical housekeeping genes belonged to the same clonal complex (CC). SCC*mec* types were identified using specific gene fragments of SCC*mec*I-V described by Oliveira et al. (30) and Hanssen and Sollid (31). The *agr* gene of each strain was compared with four genes in the NCBI GenBank database: *agr*I (GenBank accession no. AF210055), *agr*II (GenBank accession no. AF001782), *agr*III (GenBank accession no. AF001783), and *agr*IV (GenBank accession no. AF288215). The strains were assigned to the *spa* genotypes according to the Ridom SpaServer database (http://spaserver.ridom.de/). BLAST software was used to determine the similarity and respective length between sequences. The genomes of the MRSA strains were screened for antimicrobial resistance-related genes using the CARD (https://card.mcmaster.ca/). The phylogenetic tree was integrated with molecular typing data, the hVISA phenotype, and *qacA* carriage using iTOL v.5 (https://itol.embl.de/).

### Plasmid analysis and electroporation experiments

The plasmid electroporation procedure consisted of five steps: extraction of the vancomycin-resistant *S. aureus* plasmid (VRSAp) (a typical plasmid from our clinical strains carrying *qacA*), construction of the pOS1*qacAR* plasmid, transfer of the pOS1*qacAR* plasmid into DH5α cells, electroporation of the pOS1*qacAR* plasmid into JMC1 cells, and electroporation of the JMC1 demethylated pOS1*qacAR* plasmid into recipient strains.

**Fig 3:**
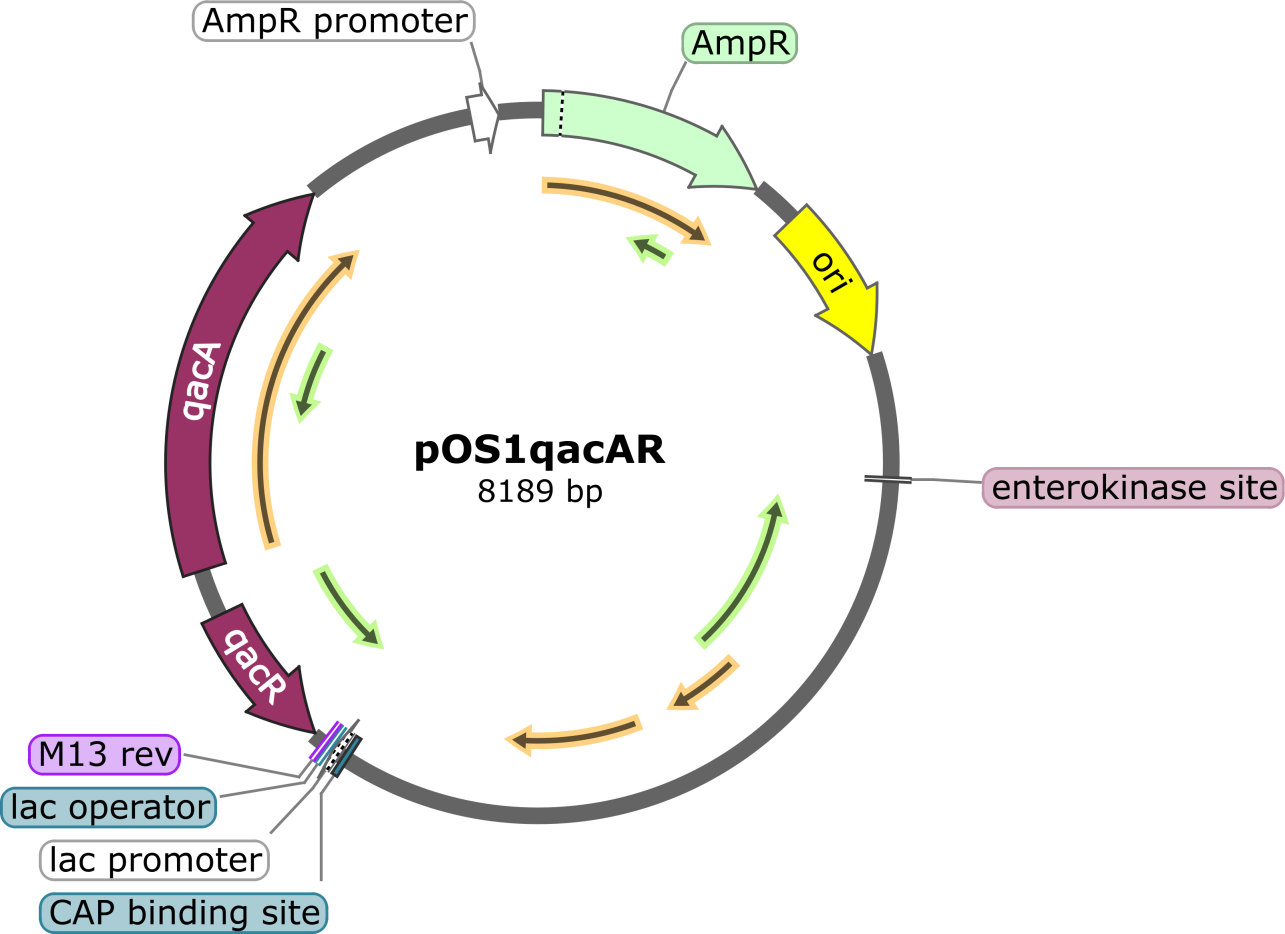
pOS1*qacAR* plasmid map.

A representative ST5-MRSA strain (serial number S1-1-76, *spa*-t2460) for which the *qacA*(+)-plasmid VRSAp was extracted using a plasmid extraction kit with the plasmid concentration determined by a NanoDrop was selected as the plasmid provider. The pOS1*qacAR* plasmid (Fig. 3) was prepared by joining the *qacAR* fragment from the VRSAp template with *qacAR*-F/*qacAR*-R as upstream and downstream PCR primers with the pOS1 plasmid fragment separated from *Escherichia coli* DH5α. We simultaneously introduced the upstream repressor protein-encoding gene *qacR* to eliminate its influence on *qacA* expression. The pOS1*qacAR* plasmid was subsequently transformed into DH5α cells, and pOS1VF/pOS1VR were used as primers for PCR and sequencing verification. Afterward, the complete pOS1*qacAR* plasmid was electroporated into the JMCI strain to obtain a demethylated pOS1*qacAR* plasmid, which is more easily accepted by recipient strains. Once the recipient strain competent cells were obtained, the demethylated pOS1*qacAR* plasmid was electroporated and subjected to PCR with the pOS1VF/pOS1VR primers, using the pOS1*qacAR* plasmid as a positive control, to obtain the corresponding *qacA* expression strains. The strains, plasmids, and primers used are summarized in Tables S2 to S4, respectively.

### Statistical analysis

All the statistical analyses were performed using SPSS 19 (SAS Institute, Cary, NC, USA). Categorical variables are presented as descriptive statistics, such as the number of cases (percent), and continuous variables are presented as medians (interquartile ranges). Univariate comparisons were performed using Fisher’s exact test for categorical variables and the Mann‒Whitney *U* test for continuous variables. *P* values less than 0.05 were considered to indicate statistical significance. All significant variables were included in the correlation analysis using GraphPad Prism 8.0 software. Variables with correlation coefficients less than 0.5 and those deemed potentially relevant to the treatment outcomes were included in the final multivariate analysis. The adequacy of the model fit was assessed using the Hosmer–Lemeshow goodness-of-fit test, with a *P* value exceeding 0.05 indicating satisfactory model fit.

## Data Availability

The complete genome sequences and annotations have been deposited in the National Center for Biotechnology Information database (https://www.ncbi.nlm.nih.gov/bioproject/) under accession number PRJNA860943. All genomic database data are also available at https://www.ncbi.nlm.nih.gov/biosample/ under accession numbers SAMN29924237–SAMN29924299 and SAMN50612176–SAMN50612229.

## SUPPLEMENTAL MATERIAL

Tables S1 to S4 spectrum.01963-25-s0001.docx Table S1: Antimicrobial resistance-related genes carriage. Table S2: MRSA strains. Table S3: Plasmids used in electroporation. Table S4: Primers used in electroporation.

ASM does not own the copyrights to Supplemental Material that may be linked to, or accessed through, an article. The authors have granted ASM a non-exclusive, world-wide license to publish the Supplemental Material files. Please contact the corresponding author directly for reuse.
