# Supplementary material for: qacA is a key factor in heteroresistance to vancomycin in sequence type 5 methicillin-resistant Staphylococcus aureus isolates from pneumonia patients
Source: Microbiol Spectr. 2026 Feb 2;14(3):e01963-25. doi: 10.1128/spectrum.01963-25 (PMC12955384; doi:10.1128/spectrum.01963-25)
Supplement: Tables S1 to S4 — Table S1: Antimicrobial resistance-related genes carriage. Table S2: MRSA strains. Table S3: Plasmids used in electroporation. Table S4: Primers used in electroporation. [file spectrum.01963-25-s0001.docx]

Table S1. Antimicrobial resistance related genes carriage in ST5-MRSA and ST764-MRSA isolates

| **No.** | **Gene** | **Total**  **(n=73)** | **ST5-MRSA (n=31)** | **ST764-MRSA (n=42)** | ***P* value** |
| --- | --- | --- | --- | --- | --- |
| 1 | *menA-lysocin* | 73 (100%) | 31 (100%) | 42 (100%) | NA |
| 2 | *tet(38)* | 73 (100%) | 31 (100%) | 42 (100%) | NA |
| 3 | *glpT* | 73 (100%) | 31 (100%) | 42 (100%) | NA |
| 4 | *mepA-MATE* | 73 (100%) | 31 (100%) | 42 (100%) | NA |
| 5 | *mepR-MATE* | 73 (100%) | 31 (100%) | 42 (100%) | NA |
| 6 | *fusE* | 73 (100%) | 31 (100%) | 42 (100%) | NA |
| 7 | *murA* | 73 (100%) | 31 (100%) | 42 (100%) | NA |
| 8 | *cls* | 73 (100%) | 31 (100%) | 42 (100%) | NA |
| 9 | *rpsL* | 73 (100%) | 31 (100%) | 42 (100%) | NA |
| 10 | *rpoB* | 73 (100%) | 31 (100%) | 42 (100%) | NA |
| 11 | *rpoC* | 73 (100%) | 31 (100%) | 42 (100%) | NA |
| 12 | *fusA* | 73 (100%) | 31 (100%) | 42 (100%) | NA |
| 13 | *EF-Tu* | 73 (100%) | 31 (100%) | 42 (100%) | NA |
| 14 | *mprF* | 73 (100%) | 31 (100%) | 42 (100%) | NA |
| 15 | *parC* | 73 (100%) | 31 (100%) | 42 (100%) | NA |
| 16 | *parE* | 73 (100%) | 31 (100%) | 42 (100%) | NA |
| 17 | *walK* | 73 (100%) | 31 (100%) | 42 (100%) | NA |
| 18 | *gyrA* | 73 (100%) | 31 (100%) | 42 (100%) | NA |
| 19 | *gyrB* | 73 (100%) | 31 (100%) | 42 (100%) | NA |
| 20 | *dfrC* | 73 (100%) | 31 (100%) | 42 (100%) | NA |
| 21 | *arlR* | 73 (100%) | 31 (100%) | 42 (100%) | NA |
| 22 | *arlS* | 73 (100%) | 31 (100%) | 42 (100%) | NA |
| 23 | *ileS* | 73 (100%) | 31 (100%) | 42 (100%) | NA |
| 24 | *mecA-PBP2* | 73 (100%) | 31 (100%) | 42 (100%) | NA |
| 25 | *ermA* | 73 (100%) | 31 (100%) | 42 (100%) | NA |
| 26 | *norA* | 72 (98.6%) | 31 (100%) | 41 (97.6%%) | >0.999 |
| 27 | *rpsJ* | 72 (98.6%) | 30 (96.8%) | 42 (100%) | 0.425 |
| 28 | *mecl-PBP2* | 72 (98.6%) | 30 (96.8%) | 42 (100%) | 0.425 |
| 29 | *ANT(9)-la* | 71 (97.3%) | 31 (100%) | 40 (95.2%) | 0.505 |
| 30 | *uhpt* | 70 (95.9%) | 28 (90.3%) | 42 (100.0%) | 0.072 |
| 31 | *fosB3* | 67 (91.8%) | 29 (93.5%) | 38 (90.5%) | 0.967 |
| 32 | *mgrA* | 63 (86.3%) | 27 (87.1%) | 36 (85.7%) | >0.999 |
| 33 | *pgsA* | 63 (86.3%) | 27 (87.1%) | 36 (85.7%) | >0.999 |
| 34 | *AAC(6’)-le-APH(2’’)-la* | 49 (67.1%) | 24 (77.4%) | 25 (59.5%) | 0.108 |
| 35 | *tet(K)* | 14 (19.2%) | 9 (29.0%) | 5 (11.9%) | 0.066 |
| 36 | *mupA* | 5 (6.8%) | 0 (0%) | 5 (11.9%) | 0.068 |
| 37 | *fusC* | 2 (2.7%) | 0 (0%) | 2 (4.8%) | 0.505 |
| 38 | *cat7A* | 1 (1.4%) | 0 (0%) | 1 (2.4%) | >0.999 |

NA：not applicable

Table S2. MRSA strains used in electroporation

| ST type | Strain Name | Description | Source |
| --- | --- | --- | --- |
| ST5 | S1-1-76 | *qacA*(+), hVISA | Clinical isolates from this study |
| ST5 | S1-2-32 | *qacA*(-), VSSA | Clinical isolates from this study |
|  | S1-2-32*qacA* | *qacA*(+), hVISA | Constructed in this study |
| ST764 | S1-2-46 | *qacA*(-), VSSA | Clinical isolates from this study |
|  | S1-2-46*qacA* | *qacA*(+), hVISA | Constructed in this study |

Table S3. Plasmids used in electroporation

| Name | Description | Source |
| --- | --- | --- |
| pOS1A | plasmid expressed in *S. aureus* | Preserved in our laboratory |
| VRSAp | *qacA*-borne plasmid from S1-1-76 strain | Clinical isolates from this study |
| pOS1*qacAR* | *qacA*(+)-plasmid carrying regulator gene *qacR* and its promotor | Constructed in this study |

Table S4. Primers used in electroporation

| Name | Sequence (5'–3') |
| --- | --- |
| *qacAR*-F | GCTGCAGGTCGACGGATCCTTATTTACTAAGTCCAT |
| *qacAR*-R | AGACGATCCGGGGAATTCCTATTTTTGTTTAGTTAT |
| pOS1VF | CACACAGGAAACAGCTATGACC |
| pOS1VR | AACTGAAGAACAACGTAACGGC |
